# Distal Versus Total D2-Gastrectomy for Gastric Cancer: a Secondary Analysis of Surgical and Oncological Outcomes Including Quality of Life in the Multicenter Randomized LOGICA-Trial

**DOI:** 10.1007/s11605-023-05683-z

**Published:** 2023-06-20

**Authors:** Cas de Jongh, Arjen van der Veen, Lodewijk A. A. Brosens, Grard A. P. Nieuwenhuijzen, Jan H. M. B. Stoot, Jelle P. Ruurda, Richard van Hillegersberg, LOGICA Study Group, Hylke J. F. Brenkman, Maarten F. J. Seesing, Misha D. P. Luyer, Jeroen E. H. Ponten, Juul J. W. Tegels, Karel W. E. Hulsewe, Bas P. L. Wijnhoven, Sjoerd M. Lagarde, Wobbe O. de Steur, Henk H. Hartgrink, Ewout A. Kouwenhoven, Marc J. van Det, Eelco Wassenaar, P. van Duijvendijk, Werner A. Draaisma, Ivo A. M. J. Broeders, Susanne S. Gisbertz, Donald L. van der Peet, Hanneke W. M. van Laarhoven

**Affiliations:** 1 Department of Surgery, University Medical Center (UMC) Utrecht, G04.228, 3508 GA Utrecht, The Netherlands; 2 Department of Pathology, University Medical Center Utrecht, Utrecht, The Netherlands; 3 Department of Surgery, Catharina Hospital Eindhoven, Eindhoven, The Netherlands; 4 Department of Surgery, Zuyderland Medical Center, Sittard, The Netherlands

**Keywords:** Gastric cancer, Gastrectomy, Postoperative complications, Quality of life, Patient selection

## Abstract

**Background:**

Distal gastrectomy (DG) for gastric cancer can cause less morbidity than total gastrectomy (TG), but may compromise radicality. No prospective studies administered neoadjuvant chemotherapy, and few assessed quality of life (QoL).

**Methods:**

The multicenter LOGICA-trial randomized laparoscopic versus open D2-gastrectomy for resectable gastric adenocarcinoma (cT1–4aN0–3bM0) in 10 Dutch hospitals. This secondary LOGICA-analysis compared surgical and oncological outcomes after DG versus TG. DG was performed for non-proximal tumors if R0-resection was deemed achievable, TG for other tumors. Postoperative complications, mortality, hospitalization, radicality, nodal yield, 1-year survival, and EORTC-QoL-questionnaires were analyzed using *Χ*^2^-/Fisher’s exact tests and regression analyses.

**Results:**

Between 2015 and 2018, 211 patients underwent DG (*n* = 122) or TG (*n* = 89), and 75% of patients underwent neoadjuvant chemotherapy. DG-patients were older, had more comorbidities, less diffuse type tumors, and lower cT-stage than TG-patients (*p* < 0.05). DG-patients experienced fewer overall complications (34% versus 57%; *p* < 0.001), also after correcting for baseline differences, lower anastomotic leakage (3% versus 19%), pneumonia (4% versus 22%), atrial fibrillation (3% versus 14%), and Clavien-Dindo grading compared to TG-patients (*p* < 0.05), and demonstrated shorter median hospital stay (6 versus 8 days; *p* < 0.001). QoL was better after DG (statistically significant and clinically relevant) in most 1-year postoperative time points. DG-patients showed 98% R0-resections, and similar 30-/90-day mortality, nodal yield (28 versus 30 nodes; *p* = 0.490), and 1-year survival after correcting for baseline differences (*p* = 0.084) compared to TG-patients.

**Conclusions:**

If oncologically feasible, DG should be preferred over TG due to less complications, faster postoperative recovery, and better QoL while achieving equivalent oncological effectiveness.

**Mini-abstract:**

Distal D2-gastrectomy for gastric cancer resulted in less complications, shorter hospitalization, quicker recovery and better quality of life compared to total D2-gastrectomy, whereas radicality, nodal yield and survival were similar.

**Supplementary Information:**

The online version contains supplementary material available at 10.1007/s11605-023-05683-z.

## Introduction 

Gastric cancer is the third leading cause of cancer-related mortality worldwide.^[1]^ Standard curative treatment consists of D2-gastrectomy combined with perioperative FLOT-chemotherapy in most countries, resulting in approximately 36–45% 5-year survival.^[2–5]^ When determining the optimal surgical strategy for gastric cancer patients, accurate patient selection is crucial to safeguard oncological effectiveness. Gastric cancer limited to the distal or middle stomach is treated with distal (D2-)gastrectomy (DG), whereas total (D2-)gastrectomy (TG) is often required for gastric cancer located in the corpus, fundus, gastric cardia, or diffusely located, and for advanced disease stages or diffuse type tumors.^[6]^

Although both DG and TG are safe to perform, previous studies exposed several differences and concerns regarding their associated surgical and oncological outcomes.^[7–11]^ DG may be associated with less morbidity, lower mortality, and better quality of life (QoL) compared to TG.^[7–10]^ Furthermore, DG could be a sound alternative for TG for older patients with substantial comorbidities and reduced performance status. On the other hand, although overall survival may be comparable if stratified for disease stage, performing DG could compromise the proximal resection margin, especially for diffuse type tumors, and the remnant stomach is at risk for developing a new primary or secondary gastric malignancy.^[7,11]^

However, no prospective studies compared outcomes after neoadjuvant chemotherapy and few assessed lymph node retrieval or quality of life, of which none in Western populations. Furthermore, there is heterogeneity among studies, most studies were retrospective and did not address the surgeon’s experience, and not all studies reported follow-up. Moreover, mainly Eastern populations were investigated, who differ from Western patients in disease stage, age, comorbidities, and BMI. Therefore, the aim of the present study was to determine the role of DG and TG for Western gastric cancer patients, in particular after neoadjuvant chemotherapy, by comparing surgical and oncological outcomes including survival and quality of life after distal versus total D2-gastrectomy in the multicenter LOGICA-cohort.

## Methods

### Study Design

This study is a secondary analysis in the prospective, multicenter LOGICA-trial to compare surgical and oncological outcomes including QoL after DG versus TG for resectable gastric adenocarcinoma (cT1–4aN0–3bM0) in 10 Dutch hospitals. The randomized controlled LOGICA-trial (NCT02248519) compared laparoscopic versus open D2-gastrectomy and showed no significant differences in surgical nor oncological outcomes including QoL. The study protocol and results were published previously.^[12,13]^ The LOGICA-trial was approved by institutional review boards at each participating center, and written consent was obtained for all patients.

### Patient Selection and Randomization

The inclusion and exclusion criteria were listed in the LOGICA protocol.^[12]^ In the LOGICA-trial, patients were randomly assigned to laparoscopic or open gastrectomy with a 1:1-ratio, and in the randomization procedure was stratified for extent of resection (DG or TG) and hospital of surgical treatment. In the current secondary analysis, all LOGICA-patients who underwent DG or TG with en-bloc D2-lymphadenectomy were included. Hence, patients without surgical resection, other surgery than DG/TG, and without D2-lymphadenectomy were excluded.

### Staging and Treatment

Regional multidisciplinary tumor boards determined the staging and individual treatment strategy according to Dutch national guidelines, which is elaborated in the LOGICA-protocol.^[4,12]^ Perioperative chemotherapy was recommended to all patients with advanced gastric cancer (cT3–4 and/or cN +) who were medically and physically fit to undergo this.

Consecutively, DG was performed for distal (pylorus, antrum) and middle (distal corpus) gastric cancer, whereas tumors located in the gastric cardia (Siewert type II/III according to TNM-8), fundus, upper corpus, or entire stomach were resected by TG.^[14]^ The surgical procedures including gastrectomy, D2-lymphadenectomy according to the Japanese Gastric Cancer Association (JGCA), complete omentectomy, and Roux-en-Y-reconstruction are described in the LOGICA study protocol.^[12,15]^ Postoperative treatment protocols were described previously and based on enhanced recovery after surgery.^[12]^ Postoperative complications were defined according to the Esophagectomy Complications Consensus Group (ECCG) and graded following the Clavien-Dindo classification.^[16,17]^ The histopathological resection specimen was evaluated according to the Dutch national guidelines and JGCA-classification.^[4,15]^

### Surgeon Experience and Quality Control

Prior to the trial start, all surgeons completed the European Society for Surgical Oncology (ESSO) Training Program on Minimally Invasive Gastrectomy and at least two laparoscopic gastrectomies per surgeon were centrally reviewed by study proctors (RvH and JR).^[18]^ Furthermore, each center performed ≥ 20 gastrectomies annually and was experienced in open gastrectomy and proficient in laparoscopic gastrectomy (≥ 20 laparoscopic cases per surgeon) for both DG and TG.

The LOGICA-trial included a mandatory surgical quality control, which comprised central assessment of intraoperatively taken photographs from the performed D2-lymphadenectomy, for which feedback was prospectively provided to participating surgeons on weekly basis.^[13]^ Additionally, the LOGICA-protocol mandated that lymph node station nos. 1–7 were clearly marked at the resection specimen at the back-table in the operating room and station nos. 8, 9, 11p/11d, and 12a were collected in separate pathology containers.

### Outcome Measures

The main outcome was overall postoperative complication rate after DG versus TG. In addition, both groups were compared regarding individual complications (i.e., anastomotic leakage, pneumonia, atrial fibrillation, ileus, abscess, pancreatic injury, chyle leakage, wound infection), mortality, oncological outcomes (e.g., radicality, marginal distances, lymph node yield, 1-year survival), intraoperative details (i.e., blood loss, operating time, conversion, additional organ resections), postoperative recovery (e.g., hospitalization, time to flatus and first oral intake, readmissions), and 1-year quality of life using EORTC QLQ-C30- and STO-22-questionnaires at baseline and after 6 weeks, 3, 6, 9, and 12 months.^[19–21]^ Furthermore, a cognitive workload questionnaire (The Subjective Mental Effort Questionnaire; SMEQ) was completed by surgeons immediately after surgery.^[22]^

### Statistical Analysis

Outcomes were compared using the (independent sample) unpaired *T*-test or Mann–Whitney *U*-test depending on the data distribution. Categorical values were compared using Fisher’s exact (if ≥ 25% of values numbered ≤ 5) or *Χ*^2^-tests. Kaplan Meier curves were plotted for survival and compared with the log-rank test. Multivariable Cox proportional hazards model was utilized to analyze overall survival, and Poisson regression with robust error variances was applied to compare overall complications after DG versus TG, and in both analyses was adjusted for the baseline differences in age, comorbidities, disease stage, and histological subtype.^[23,24]^ The time period for survival was time in days from inclusion to death due to any reason. QoL was compared using linear mixed-effects regression, adjusting for baseline-QoL and stratifying for hospital of surgical treatment. Differences in QoL-scores were presented with 95% confidence intervals (CI) and categorized in trivial, small, medium, or large differences for each individual QoL-subscale separately according to previous QoL-guidelines to assess their clinical relevance.^[25,26]^ A two-sided *p* < 0.05 was considered statistically significant for all tests, which were performed by IBM SPSS Statistics version 27.0 (SPSS Inc. Chicago, USA).

## Results

**Fig. 1:**
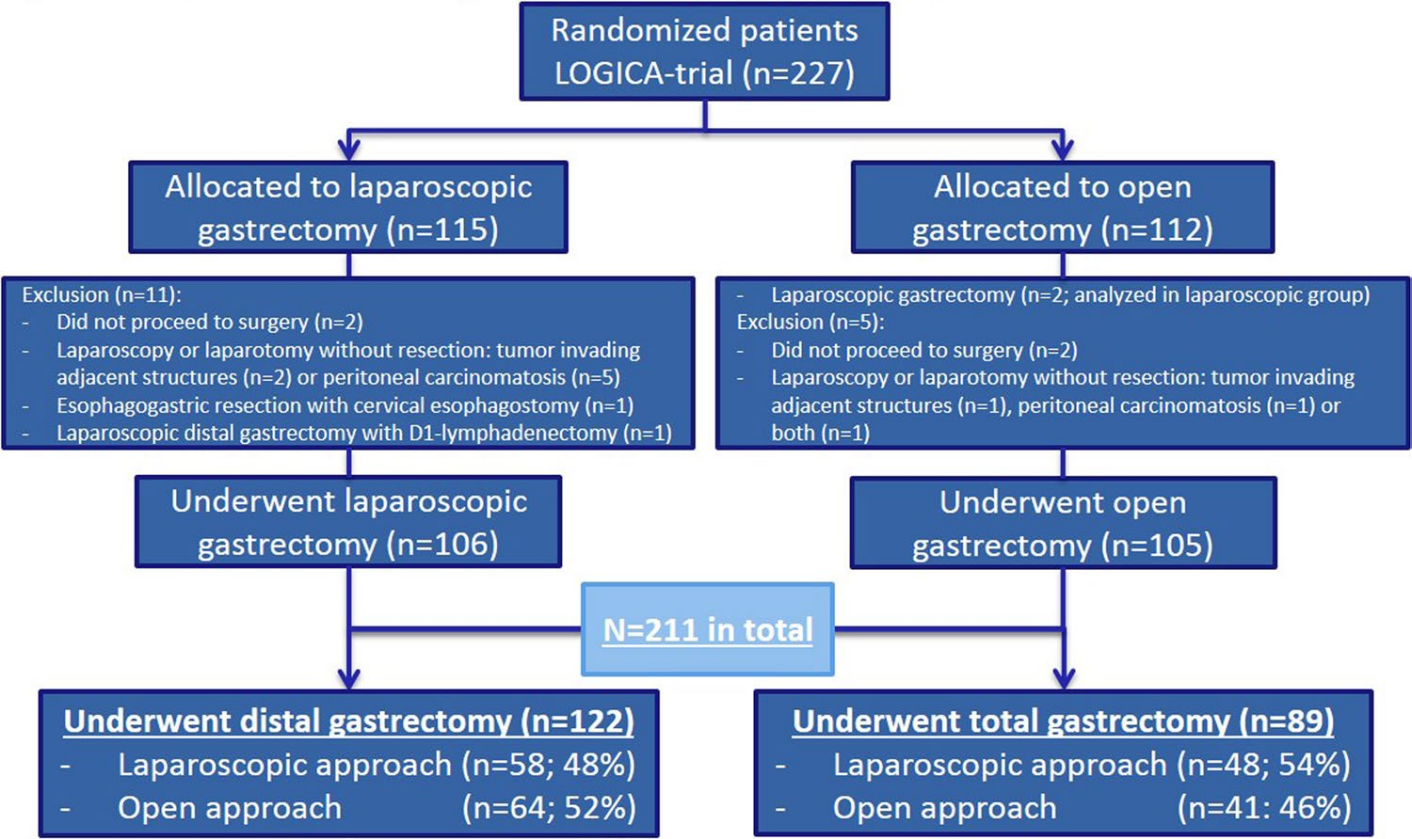
Study flow chart of the 211 included patients in the current study

Between 2015 and 2018, 211 of the 227 LOGICA-patients (93%) were included (Fig. 1) and underwent DG (*n* = 122) or TG (*n* = 89). Patients were excluded if they did not undergo surgical resection of the tumor (*n* = 14), or underwent esophagogastric resection (*n* = 1) or D1 + /D1-lymphadenectomy (*n* = 1).

The baseline characteristics are presented in Table 1. Patients undergoing DG were older (69 versus 66 years; *p* = 0.041) and had more comorbidities (88% versus 74%; *p* = 0.019), more distal tumors (77% versus 30%;* p* < 0.001), less diffuse type tumors (31% versus 51%; *p* = 0.005), and lower clinical T-stage (*p* = 0.001) compared to patients undergoing TG.

**Table 1:** Baseline characteristics

Baseline characteristics	Distal gastrectomy (*n* = 122)	Total gastrectomy (*n* = 89)	*p*-value
Entire cohort: *n* = 211 (100%)			
Age (in years; mean [SD])	69 [± 9.9]	66 [± 11.5]	***0.041***
Male sex	82 (67)	50 (56)	*0.136*
BMI, kg/m^2^ (median [IQR])	25.6 [23.2–28.7]	25.3 [22.3–28.0]	*0.382*
ECOG-performance status* 0 1 2	53 (51) 45 (43) 6 (6)	39 (49) 35 (44) 6 (8)	*0.933*
Weight loss^¥^ No Yes	50 (44) 64 (56)	36 (42) 49 (58)	*0.797*
ASA-classification^#^ 1 2 3	11 (9) 80 (66) 31 (25)	9 (10) 59 (66) 21 (24)	*0.933*
Any comorbidity	107 (88)	66 (74)	***0.019***
Cardiovascular comorbidity	75 (61)	44 (49)	*0.109*
Pulmonary comorbidity	23 (19)	21 (24)	*0.505*
Gastrointestinal comorbidity	34 (28)	22 (25)	*0.723*
Previous abdominal surgery	34 (28)	24 (28)	*1*
Tumor location Proximal stomach Middle stomach Distal stomach	0 (0) 28 (23) 94 (77)	22 (25) 40 (45) 27 (30)	** < *0.001***
Lauren classification Intestinal type Diffuse type	85 (70) 37 (30)	45 (51) 44 (49)	***0.005***
Clinical *T*-stage cT1 cT2 cT3 cT4	12 (10) 45 (37) 58 (48) 7 (6)	2 (2) 17 (19) 59 (66) 11 (12)	***0.001***
Clinical *N*-stage; cN1–3	53 (43)	43 (48)	*0.574*
Neoadjuvant chemotherapy	87 (71)	70 (79)	*0.240*

Percentages were calculated after excluding missing values and may not add up to 100% due to rounding

Bold numbers indicate statistical significance

*SD* standard deviation, *BMI* body mass index (kg/m^2^), *IQR* interquartile range, *ECOG* Eastern Cooperative Oncology Group, *ASA* American Society of Anesthesiologists

*There were 18 (15%) and 9 (10%) missing values for the distal and total gastrectomy group regarding ECOG-performance status

^¥^There were 8 (7%) and 4 (4%) missing values for the distal and total gastrectomy group regarding weight loss

^#^There were 4 (3%) and 9 (10%) missing values for the distal and total gastrectomy group regarding ASA-classification

Neoadjuvant chemotherapy was administered to 157 of the 211 patients (74%) in similar proportions for DG- versus TG-patients (71% versus 79%; *p* = 0.240), and consisted of the MAGIC-regimen or an equivalent regimen (*n* = 120/157; 76%), FLOT-regimen (*n* = 28/157; 18%), or other regimens (*n* = 9/157; 6%).

### Intraoperative Data

Intraoperative details are listed in Table 2. All patients (*n* = 211; 100%) underwent D2-lymphadenectomy and Roux-en-Y reconstruction. Jejunal pouch reconstruction was performed in 18% of TG-patients. Jejunal feeding tube placement (7% versus 30%; *p* < 0.001) was less often performed after DG than TG. The proportion laparoscopic resections was comparable regarding DG versus TG (48% versus 54%; *p* = 0.437). Unplanned splenectomy was performed less frequently during DG versus TG (0% versus 4%; *p* = 0.030), whereas pancreatectomy rate was not different (0% versus 2%; *p* = 0.177). DG led to significantly shorter operating time (mean 196 versus 218 min; *p* = 0.007) and less blood loss (median 200 versus 300 mL; *p* = 0.001) compared to TG, whereas intraoperative complications were similar (*p* = 0.230). The surgeon mental effort between DG and TG differed but did not reach statistical significance (53.9 versus 60.1; *p* = 0.087).

**Table 2:** Intraoperative details for distal versus total gastrectomy

Intraoperative details	Distal gastrectomy (*n* = 122)	Total gastrectomy (*n* = 89)	*p*-value
Entire cohort: *n* = 211 (100%)			
Laparoscopic gastrectomy	58 (48)	48 (54)	*0.437*
D2-lymphadenectomy	122 (100)	89 (100)	*1*
Roux-en-Y reconstruction	122 (100)	89 (100)	*1*
Jejunal pouch reconstruction	-	16 (18)	
Jejunal feeding tube	8 (7)	27 (30)	** < *0.001***
Splenectomy	0 (0)	4 (5)	***0.030***
Pancreatectomy	0 (0)	2 (2)	*0.177*
Operating time, *minutes* (mean [SD])	196 [± 57]	218 [± 59]	***0.007***
Blood loss, *millilitre* (median [IQR])	200 [100–343]	300 [150–600]	***0.001***
Intraoperative complications None Bleeding Pancreatic injury Other	116 (95) 3 (2) 1 (1) 2 (2)	81 (91) 6 (7) 2 (2) 0 (0)	*0.230*
Conversion	2 (2)	5 (6)	*0.135*
Surgeon mental effort* (mean[SD])	53.9 [± 22.7]	60.1 [± 20.0]	*0.087*

Percentages may not add up to 100% due to rounding

Bold numbers indicate statistical significance

*SD* standard deviation, *IQR* interquartile range

*The Surgeon Mental Effort Questionnaire was filled out by the operating surgeon directly after completing the operation. There were missing values (at random) in the distal gastrectomy group for 49 cases (40%) and in the total gastrectomy group for 20 cases (23%)

### Postoperative Data

Postoperative complications and recovery are shown in Table 3. Overall postoperative complication rate was significantly lower after DG versus TG (34% versus 57%; *p* = 0.001). Also after correcting for confounders and for the baseline differences in age, comorbidities, histological subtype, and cT-stage using multivariable Poisson regression with robust error variances (Supplementary Table 1), there were significantly fewer overall postoperative complications after DG compared to TG (*p* < 0.001).

**Table 3:** Postoperative complications and recovery

Postoperative complications	Distal gastrectomy (*n* = 122)	Total gastrectomy (*n* = 89)	*p*-value
Entire cohort: *n* = 211 (100%)			
Overall postoperative complications	41 (34)	51 (57)	***0.001***
Clavien-Dindo grading (most severe)			***0.006***
Grade 1	9 (7)	4 (5)	
Grade 2	14 (11)	23 (26)	
Grade 3A	5 (4)	6 (7)	
Grade 3B	4 (3)	6 (7)	
Grade 4A	2 (2)	6 (7)	
Grade 4B	1 (1)	0 (0)	
Grade 5	6 (5)	6 (7)	
Anastomotic leakage	3 (2)	17 (19)	** < *0.001***
Anastomotic leakage grading (ECCG)			** < *0.001***
Grade I	0 (0)	4 (5)	
Grade II	0 (0)	2 (2)	
Grade III	3 (3)	11 (12)	
Pneumonia	9 (7)	20 (22)	***0.003***
Atrial fibrillation or flutter	5 (4)	12 (13)	***0.020***
Ileus	3 (2)	6 (7)	*0.172*
Intra-abdominal abscess	1 (1)	5 (6)	*0.085*
Pancreatitis or pancreatic fistula	4 (3)	1 (1)	*0.400*
Chyle leakage	0 (0)	3 (3)	*0.074*
Sepsis	4 (3)	4 (5)	*0.724*
Intestinal ischemia	2 (2)	3 (3)	*0.652*
Wound infection	4 (3)	5 (6)	*0.498*
Fascia dehiscence	3 (2)	1 (1)	*0.640*
Delirium	3 (2)	3 (3)	*0.698*
Feeding jejunostomy complication	1 (1)	2 (2)	*0.574*
Other complications	18 (15)	14 (16)	*0.799*
Postoperative recovery	Distal gastrectomy (n = 122)	Total gastrectomy (n = 89)	*p*-value
Time to first oral intake *days* (median [IQR])	1 [1 – 1]	1 [1 – 2]	***0.005***
Time to first defecation *days* (median [IQR])	4 [3 – 5]	4 [3 – 5]	***0.003***
Discharge criteria fulfilled *days* (median [IQR])	6 [4 – 7]	8 [6 – 11]	** < *0.001***
Hospital stay *days* (median [IQR])	6 [5 – 8]	8 [7 – 12]	** < *0.001***
Intensive Care Unit stay *days* (median [IQR])	0 [0–1]	0 [0–1]	***0.006***
In-hospital mortality	6 (5)	6 (7)	*0.566*
30-day mortality	5 (4)	4 (5)	*1*
90-day mortality	7 (6)	9 (10)	*0.356*
Readmission < 30 days after discharge	15 (12)	6 (7)	*0.272*

Percentages may not add up to 100% due to rounding

Bold numbers indicate statistical significance

*ECCG* Esophagectomy Complications Consensus Group, *IQR* interquartile range

In addition, anastomotic leakage (3% versus 19%; *p* < 0.001), pneumonia (7% versus 23%; *p* = 0.003), atrial fibrillation (4% versus 14%; *p* = 0.020), and the severity of overall complications illustrated in Clavien-Dindo grading (*p* = 0.006) and anastomotic leakage illustrated in ECCG-grading (*p* < 0.001) were significantly lower in favor of DG compared to TG.^[16,17]^ Other complications and 30-/90-day mortality rates were similar for both groups (*p* > 0.05).

The median number of days were significantly different in favor of DG-patients compared to TG-patients regarding time to first oral intake (90% versus 70% within 1 day; *p* = 0.005), time to first defecation (73% versus 55% within 4 days; *p* = 0.003), meeting discharge criteria (6 versus 8 days; *p* < 0.001), hospital stay (6 versus 8 days; *p* < 0.001), and intensive care unit (ICU) stay (88% versus 73% no ICU-admission at all; *p* = 0.006). Readmission rates within 30 days after discharge did not differ for both groups (12% versus 7%; *p* = 0.272).

Regarding surgical approach (laparoscopic versus open), there were no significant differences in postoperative complications, hospitalization, or postoperative recovery, which was further elucidated in detail in the previously published LOGICA-trial main results.^[13]^

### Oncological Outcomes

Histopathological results are listed in Table 4. Patients selected for DG had 98% R0-resection rate (*n* = 120/122). After TG, R0-resection rate (91%; *n* = 81/89) was lower (*p* = 0.019), but the TG-group had larger tumor diameter (55 versus 35 mm; *p* = 0.023), higher clinical T-stages (*p* = 0.001), and more diffuse type tumors (51% versus 31%; *p* = 0.005). Hence, after correcting for these variables and confounders using multivariable logistic regression (Supplementary Table 2), the resection margin status between both groups was similar (*p* = 0.264).

**Table 4:** Histopathological results after distal versus total gastrectomy

Histopathological results	Distal gastrectomy (*n* = 122)	Total gastrectomy (*n* = 89)	*p*-value
Entire cohort: *n* = 211 (100%)			
Tumor histology			*1*
Adenocarcinoma	121 (99)	88 (99)	
Neuroendocrine tumor/carcinoma	1 (1)	1 (1)	
Lymph node yield (median [IQR])	28 [21 – 38]	30 [22 – 37]	*0.490*
Radicality; R0-resections	120 (98)	81 (91)	***0.019***
Distance (mm) to proximal margin (median [IQR])	50 [19–80]	28 [10–60]	***0.002***
Distance (mm) to distal margin (median [IQR])	25 [10 – 40]	40 [10–77]	***0.030***
Maximum tumor diameter (mm) (median [IQR])	35 [20–55]	55 [35–91]	***0.023***
Pathological stage	10 (8)		*0.089*
(y)pT0	20 (16)	4 (4)	
(y)pT1/Tis	18 (15)	10 (11)	
(y)pT2	46 (38)	8 (9)	
(y)pT3	28 (23)	41 (46)	
(y)pT4a	0 (0)	22 (25)	
(y)pT4b		4 (4)	
Nodal stage			*0.644*
(y)pN0	56 (46)	38 (43)	
(y)pN +	66 (54)	51 (57)	
Mandard tumor regression grading			*0.400*
Grade 1	10 (8)	4 (4)	
Grade 2	6 (5)	2 (2)	
Grade 3	28 (23)	20 (22)	
Grade 4	23 (19)	22 (25)	
Grade 5	19 (16)	21 (24)	
No neoadjuvant treatment	36 (30)	20 (22)	

Percentages may not add up to 100% due to rounding

Bold numbers indicate statistical significance

*IQR* interquartile range, *mm* millimeter

Positive resection margins after DG (*n* = 2) were both diffuse type (distal) tumors extending into the proximal margin. After TG, the positive margins (*n* = 8) were either proximal (*n* = 4) or both proximal and distal (*n* = 4), of whom 7 patients (88%) had diffuse type tumors, and these 8 tumors were located proximal (*n* = 1), middle (*n* = 3), and distal (*n* = 4). Proximal margin distances were larger after DG (50 versus 28 mm; *p* = 0.002) as DG-patients had more distal tumors (77% versus 30%), while TG-patients had more proximal tumors (0% versus 25%) resulting in larger distances to distal margins (25 versus 40 mm; *p* = 0.030).

When evaluating resection margin status in subgroups (Supplementary Table 3), R1-resections occurred more frequently for diffuse versus intestinal type tumors for both DG-patients (95% versus 100% R0) and TG-patients (84% versus 98% R0). Regarding cT3-4- versus cT1-2-stage, more R1-resections were found for TG-patients (89% versus 100% R0), but not for DG-patients (98% versus 98% R0). After neoadjuvant chemotherapy (yes versus no), the resection margin status was similar after both DG (yes; 98% versus no; 100%) and TG (yes; 91% versus no; 90%). In multivariable regression analyses (Supplementary Table 2), diffuse type tumors were independently associated with positive resection margins (OR 10.04; *p* = 0.035), whereas cT-stage (OR 2.76; *p* = 0.371) and neoadjuvant chemotherapy (OR 1.03; *p* = 0.973) were not.

Median lymph node yield (28 versus 30 nodes; *p* = 0.490), (y)pT-stage (*p* = 0.089), and Mandard tumor regression grading (*p* = 0.400) were similar in both groups (*p* > 0.05).

Overall survival analyses are displayed in Table 5. Univariate analysis and Kaplan Meier curves (Supplementary Fig. 1) showed better overall survival for the DG- versus TG-group (*p* < 0.05), but did not incorporate differences in baseline. Hence, after adjusting for the baseline differences in age, comorbidities, tumor location, histological subtype, and cT-stage in multivariable analyses, overall survival for patients undergoing DG versus TG was not significantly different (*p* = 0.084). The only independent predictor for overall survival was administration of neoadjuvant chemotherapy (HR 0.41 [95% CI 0.20–0.87]; *p* = 0.020).

**Table 5:** Overall survival of the distal versus total gastrectomy patient group using Cox regression

Cox proportional hazards model Entire cohort: *n* = 211 patients (100%)	Univariable		Multivariable	
	HR [95% CI]	*p*-value	HR [95% CI]	*p*-value
Age (per year)	1.00 [0.97–1.03]	*0.805*	1.00 [0.96–1.03]	*0.796*
Any comorbidity (yes)	0.87 [0.40–1.90]	*0.734*	1.13 [0.50–2.55]	*0.763*
Tumor location Proximal stomach Middle stomach Distal stomach	– 1.52 [0.51–4.49] 0.81 [0.28–2.40]	*–* *0.450* *0.706*	– 1.77 [0.58–5,44] 1.21 [0.37–3.95]	*–* *0.320* *0.757*
Extent of surgical resection Distal gastrectomy Total gastrectomy	– **2.23 [1.19–4.20]**	*–* ***0.013***	– 1.94 [0.92–4.12]	*–* *0.084*
Lauren classification Intestinal type Diffuse type	– 1.62 [0.87–3.02]	*–* *0.126*	– 1.45 [0.72–2.91]	*–* *0.295*
Clinical T-stage cT1–2 cT3–4	– 1.56 [0.78–3.12]	*–* *0.210*	– 1.58 [0.75–3.30]	*–* *0.227*
Neoadjuvant chemotherapy (yes)	**0.52 [0.28–0.99]**	***0.048***	**0.41 [0.20–0.87]**	***0.020***

In this multivariable Cox proportional hazards model, age, any comorbidity, tumor location, cT-stage, and Lauren histological subtype were included in the model due to the significant differences in baseline characteristics between the distal and total gastrectomy groups. In supplementary material, the Kaplan Meier curves are displayed, but these plots are not adjusted for the baseline differences

Bold numbers indicate statistical significance

*HR* hazard ratio, *95% CI* 95% confidence interval

### Quality of Life

[]The QoL differences reported by patients undergoing DG versus TG postoperatively at 6 weeks and 3, 6, 9, and 12 months are shown in Table 6. After correcting for baseline QoL and hospital of surgical treatment in the linear mixed-effects regression, QoL was significantly better after DG for global health, in 6 out of 7 functional scales, and for 13 of the 17 symptom scales during at least one or all time points [95% CI did not include 0 points difference]. When assessing clinical relevance, most significantly different QoL-values ranged ≥ 10 points favoring DG compared to TG with regard to global health, fatigue, nausea and vomiting, dysphagia, pain, reflux, insomnia, appetite loss, eating restrictions, diarrhea, role functioning, body image, anxiety, dry mouth, and taste. At all time points, the significant QoL-differences were also clinically relevant, categorized in either medium (41%) or small (59%) differences, without any trivial (0%) differences.^[25,26]^

[Edit]

**Table 6:** Quality of life differences after distal versus total D2-gastrectomy using EORTC QLQ-C30 (top) and STO-22 (bottom) questionnaires

	Distal gastrectomy group at baseline (preoperative)	Distal vs total gastrectomy at 6 weeks	Distal vs total gastrectomy at 3 months	Distal vs total gastrectomy at 6 months	Distal vs total gastrectomy at 9 months	Distal vs total gastrectomy at 1 year
	Mean (± SD)^3^	Mean [95% CI]^3^	Mean [95% CI]^3^	Mean [95% CI]^3^	Mean [95% CI]^3^	Mean [95% CI]^3^
Quality of life questionnaire (QLQ)-C30						
Global health–related quality of life^1^	69 [22]	+ 4.1 [− 2.1 to 10.4]	+ 10.5^*^ [4.3 to 16.7]	** + 7.7**^£^ **[1.3 to 14.0]**	+ 11.8^*^ [5.2 to 18.3]	+ 6.4 [− 0.4 to 13.1]
*Functional scales*^*1*^						
Physical functioning	81 [18]	** + 6.3**^£^ **[0.6 to 12.0]**	+ 4.1 [− 1.5 to 9.8]	+ 2.2 [− 3.5 to 8.0]	+ 5.4 [− 0.5 to 11.3]	+ 4.8 [− 1.3 to 10.8]
Role functioning	72 [28]	** + 11.0**^£^** [2.5 to 19.6]**	** + 13.2**^£^** [4.8 to 21.7]**	** + 10.2**^£^** [1.6 to 18.8]**	** + 10.5**^£^** [1.6 to 19.4]**	** + 10.6**^£^** [1.4 to 19.8]**
Emotional functioning	80 [22]	− 1.6 [− 7.6 to 4.4]	+ 5.5 [− 0.5 to 11.5]	+ 4.3 [− 1.8 to 10.4]	+ 4.7 [− 1.6 to 11.1]	+ 4.2 [− 2.3 to 10.7]
Cognitive functioning	88 [17]	− 0.1 [− 6.0 to 5.9]	** + 6.6**^£^ **[0.7 to 12.6]**	+ 2.3 [− 3.8 to 8.4]	+ 4.3 [− 2.0 to 10.6]	+ 6.0 [− 0.4 to 12.5]
Social functioning	79 [26]	** + 7.4**^£^ **[0.55 to 14.3]**	** + 8.1**^£^ **[1.3 to 15.0]**	+ 5.1 [− 1.9 to 12.1]	** + 9.1**^£^ **[1.9 to 16.4]**	** + 7.8**^£^ **[0.3 to 15.3]**
*Symptom scales*^*2*^						
Fatigue	32 [25]	** − 9.7**^£^ **[− 17.1 to –2.4]**	** − 8.8**^£^ **[− 16.2 to − 1.5]**	** − 12.3**^£^ **[− 19.8 to − 4.9]**	** − 10.0**^£^ **[− 17.7 to − 2.3]**	** − 10.1**^£^ **[− 18.0 to − 2.1]**
Nausea and vomiting	10 [19]	− 8.9^*^ [− 15.7 to − 2.0]	− 11.3^*^ [− 18.1 to − 4.5]	− 13.3^*^ [− 20.3 to − 6.4]	− 6.6 [− 13.8 to 0.6]	− 2.6 [− 10.0 to 4.8]
Pain	14 [22]	− 1.8 [− 9.5 to 5.9]	− 5.2 [− 12.9 to 2.5]	− 4.4 [− 12.3 to 3.4]	− 7.7 [− 15.8 to 0.4]	− 8.0 [− 16.3 to 0.4]
Dyspnea	17 [27]	− 6.0 [− 13.1 to 1.1]	− 6.3 [− 13.4 to 0.7]	− 0.3 [− 7.5 to 6.8]	− 0.4 [− 7.8 to 7.0]	** − 7.8**^£^ **[− 15.5 to − 0.2]**
Insomnia	26 [32]	− 0.9 [− 11.1 to 9.3]	** − 11.9**^£^ **[− 22.1 to − 1.8]**	− 14.1^*^ [− 24.4 to − 3.7]	** − 11.3**^£^ **[− 21.9 to − 0.6]**	− 6.2 [− 17.1 to 4.8]
Appetite loss	20 [31]	** − 12.1**^£^ **[− 22.1 to − 2.1]**	− 17.1^*^ [− 27.1 to − 7.1]	− 23.9^*^ [− 34.1 to − 13.7]	− 14.5^*^ [− 25.1 to − 3.9]	** − 12.8**^£^ **[− 23.6 to − 1.9]**
Constipation	12 [25]	+ 2.9 [− 3.8 to 9.6]	+ 1.6 [− 5.1 to 8.3]	− 2.2 [− 9.1 to 4.7]	− 1.4 [− 8.5 to 5.7]	− 0.9 [− 8.2 to 6.4]
Diarrhea	10 [22]	− 10.9* [− 19.2 to − 2.6]	− 11.5^*^ [− 19.7 to − 3.2]	− 9.9^*^ [− 18.3 to − 1.4]	− 9.0^*^ [− 17.8 to − 0.2]	− 9.2* [− 18.2 to − 0.2]
Financial difficulties	8 [23]	+ 1.2 [− 4.5 to 7.0]	+ 3.5 [− 2.2 to 9.2]	+ 5.3 [− 0.6 to 11.1]	− 3.1 [− 9.1 to 2.9]	+ 1.3 [− 4.9 to 7.5]
Quality of life questionnaire STO-022						
*Functional scales*^*1*^						
Body image	80 [30]	+ 17.1^*^ [8.6 to 25.6]	** + 10.6**^£^** [2.2 to 19.1]**	+ 12.8^*^ [4.1 to 21.4]	** + 10.6**^£^** [1.7 to 19.1]**	+ 14.2^*^ [5.0 to 23.4]
*Symptom scales*^*2*^						
Dysphagia	14 [24]	− 14.6^*^ [− 21.1 to − 8.1]	− 16.8^*^ [− 23.3 to − 10.3]	− 17.0^*^ [− 23.6 to − 10.4]	** − 11.1**^£^ **[− 18.0 to − 4.3]**	** − 9.9**^£^ **[− 16.9 to − 2.8]**
Pain	17 [23]	** − 8.0**^£^ **[− 14.3 to − 1.8]**	** − 11.1**^£^ **[− 17.3 to − 4.9]**	** − 9.1**^£^ **[− 15.4 to − 2.7]**	** − 11.0**^£^ **[− 17.5 to − 4.4]**	** − 11.0**^£^ **[− 17.7 to − 4.3]**
Reflux	15 [22]	− 6.2 [− 12.9 to 0.6]	− 13.5^*^ [− 20.2 to − 6.7]	** − 9.1**^£^ **[− 16.0 to − 2.3]**	** − 12.8**^£^ **[− 19.9 to − 5.8]**	** − 10.4**^£^ **[− 17.6 to − 3.1]**
Eating restrictions	21 [26]	** − 12.5**^£^ **[− 19.5 to − 5.4]**	− 16.9^*^ [− 24.0 to − 9.9]	− 21.1^*^ [− 28.2 to − 13.9]	− 17.1^*^ [− 24.4 to − 9.7]	− 16.0^*^ [− 23.5 to − 8.4]
Anxiety	36 [24]	** − 9.0**^£^ **[− 16.1 to − 1.9]**	** − 12.3**^£^ **[− 19.4 to − 5.3]**	** − 11.6**^£^ **[− 18.8 to − 4.4]**	+ 14.3^*^ [− 21.8 to − 6.8]	− 14.0^*^ [− 21.7 to − 6.3]
Dry mouth	22 [29]	− 6.8 [− 16.4 to 2.7]	− 7.6 [− 17.1 to 1.9]	** − 10.4**^£^ **[− 20.2 to − 0.7]**	− 8.8 [− 18.9 to 1.3]	− 4.5 [− 14.9 to − 5.8]
Taste	27 [34]	− 1.3 [− 11.3 to 8.8]	− 5.6 [− 15.6 to 4.4]	13.3^*^ [− 23.5 to − 3.1]	− 6.2 [− 16.8 to 4.3]	− 5.7 [− 16.6 to 5.2]
Hair loss	32 [41]	− 7.0 [− 17.3 to 3.4]	+ 2.2 [− 8.3 to 12.7]	+ 1.4 [− 9.5 to 12.3]	+ 1.6 [− 9.8 to 13.0]	+ 5.1 [− 6.6 to 16.8]

Displayed differences are for distal versus total gastrectomy (i.e., plus sign indicates higher values in the distal group). Bold numbers indicate statistical significance, meaning that 95% confidence intervals of the differences between distal and total gastrectomy did not include a difference of 0. Significant differences were categorized into trivial, small (£), medium (*), or large differences according to previously published guidelines^[25,26]^

*SD* standard deviation, *CI* confidence interval

^1^Scores range, 0–100: higher scores represent a better quality of life or functioning

^2^Scores range, 0–100: higher scores represent more severe symptoms

The raw 1-year QoL-data are displayed in Supplementary Table 4 and 5. After DG, all functional and symptom scales seemed to restore to the preoperative baseline at 3 months after surgery. After TG, most items recovered generally in 6 months, and no full recovery within 12 months was found for pain, dysphagia, reflux, eating restrictions, diarrhea, and body image. For DG, global health-related QoL; role, emotional, and social functioning; pain; dysphagia; and anxiety showed median 6–17 points better QoL-values at 6–12 months than preoperatively. Such improvements were not found after TG.

The proportion of patients with preoperative weight loss was similar after DG and TG (56% versus 58%; *p* = 0.797). At 1 year postoperatively, 64% of patients (*n* = 73/115) had ≥ 2 kg weight loss, which occurred less frequently after distal than total gastrectomy (52% versus 83%; *p* = 0.003), as shown in Supplementary Table 6. Compared to preoperative weight, median weight differences at 1 year were significant (*p* < 0.001), showing − 4 kg after DG [IQR + 1 to − 8 kg] and − 10 kg after TG [− 5 to − 15 kg].

## Discussion

This study aimed to determine the role of DG for Western gastric cancer patients by comparing surgical and oncological outcomes including quality of life after DG versus TG, in particular in a population where the vast majority of patients was treated with neoadjuvant chemotherapy. Patients selected to undergo DG experienced significantly fewer and less severe postoperative complications, also after correcting for baseline differences. Furthermore, DG-patients had better intraoperative surgical outcomes, shorter hospital and ICU stay, and quicker postoperative recovery compared to TG-patients. Moreover, the reported quality of life after DG was significantly better in most functional and symptom scales at one or all time points, and the significant differences were also clinically relevant based on previous guidelines.^[25,26]^ Additionally, radicality and overall survival corrected for baseline differences were comparable between both groups, and postoperative mortality and lymph node yield were similar. These results confirm the surgical safety and oncological effectiveness of DG for Western gastric cancer patients if carefully selected based on tumor and patient characteristics, also after neoadjuvant chemotherapy and for advanced disease stage.

In the present study, both DG and TG were oncologically effective and oncological outcomes were concordant with current standards.^[2–4,27–29]^ Gastrectomy for gastric cancer is primarily aimed at achieving R0-resection, which has been correlated with prolonged survival.^[30,31]^ In the current study, TG was often required for proximal, advanced, diffuse type gastric cancer with larger tumor size to achieve radical resections. Two previous meta-analyses did not compare resection margin status between DG and TG.^[7,32]^ Although DG could theoretically compromise the proximal resection margin, our cohort presented a very good 98% R0-resection rate after DG. Additionally, this conclusion was robust to subgroup-analyses of only DG-patients for advanced disease stage (98%), diffuse type tumors (95%), and after neoadjuvant chemotherapy (98%), which is in line with a previous study.^[33]^ Furthermore, lymph node yield and overall survival stratified for disease stage and corrected for the baseline differences were comparable in both groups, as was also demonstrated in previous studies.^[7,32]^ Therefore, our results strongly support performing DG for both early and advanced gastric cancer located in the middle and/or distal stomach, also for the diffuse histological subtype and irrespective of neoadjuvant treatment, but on the essential condition that the proximal resection margin is secured. To this end, intraoperative frozen sections show low rates of false-negative outcomes (1–2.5%) and are highly recommended, especially for diffuse type and signet ring cell carcinomas which independently predicted positive resection margins in our cohort and previous studies.^[34–37]^

Importantly, our results demonstrate that DG resulted in fewer and less severe postoperative complications (overall, anastomotic leakage, pneumonia, and atrial fibrillation), less blood loss and splenectomy, shorter operating time and hospital and ICU stay, quicker postoperative recovery, and better quality of life compared to TG. This is in line with a previous nationwide evaluation.^[29]^ Additionally, a meta-analysis of 3554 patients containing mostly retrospective studies and few clinical trials, all without neoadjuvant chemotherapy, reported similar results favoring DG and showed that TG-patients suffer from higher complication and mortality rates, longer operating time, and more intraoperative blood loss.^[7]^ Hence, DG results in optimal safety of surgery due to lower perioperative morbidity, shorter hospitalization, faster postoperative recovery, and better patient-reported outcomes, both with and without neoadjuvant chemotherapy. This strongly supports performing DG after careful patient selection.

Patients in the current study experienced significantly better quality of life after DG versus TG regarding most functional and symptom scales at one or all time points in the 1-year follow-up (predominantly ≥ 10 points difference), possibly as a consequence of functional preservation of part of the stomach. Importantly, all significant differences were also clinically relevant and categorized in medium (41%) or small differences (59%) based on previous guidelines, without any trivial (0%) differences.^[25,26]^ The current Western cohort presents unique and comprehensive prospective quality of life data with substantial improvements favoring DG. Interestingly, QoL-items after DG restored to the preoperative baseline faster than after TG (± 3 versus 6–12 months). Moreover, after DG, 7 items even reached better QoL-values at 6–12 months after surgery than preoperatively, whereas after TG several symptoms (pain, reflux, eating restrictions, diarrhea) did not fully recover within 12 months. The reported quality of life scores were comparable in value to a previous nationwide evaluation, suggesting that our results are representative.^[38]^ Three previous single-center Asian studies without neoadjuvant chemotherapy assessed quality of life and presented similar conclusions.^[8–10]^ Accordingly, although TG results in acceptable quality of life, DG leads to better quality of life, also after neoadjuvant chemotherapy.

To complement the abovementioned, several recommendations for surgical decision making can be stated. The surgical strategy (DG or TG) should primarily be based upon achieving a radical D2-gastrectomy, and may secondarily be adjusted to ensure safety of surgery. It should be noted that there is not always a “choice” for surgeons between performing DG or TG; for instance, proximal tumors are not eligible for distal gastrectomy. In the current study, the differences in baseline characteristics reflect this surgical selection process. Notably, the R0-resection rates after DG and TG (98% versus 91%) should be interpreted within the context of these baseline differences, since the TG-group contained proximal tumors and had larger tumor diameters (*p* = 0.023), higher cT-stages (*p* = 0.001), and more diffuse type tumors (*p* = 0.005), which predict positive resection margins.^[30,31]^ In addition, patients selected to undergo DG were older and had more comorbidities compared to TG-patients. Since patients with older age, more comorbidities, poor performance status, and deteriorated body composition during neoadjuvant chemotherapy have been related to poorer surgical perioperative outcomes, such characteristics should also be taken into account when selecting patients for DG or TG.^[39–41]^ Furthermore, although infrequently, gastric cancer of the remnant stomach after DG can occur at long-term, which should not be neglected in the surgical decision making.^[11]^ Hence, incorporating both tumor and patient characteristics when balancing radicality, surgical risk and morbidity in order to determine the extent of resection (DG or TG) is crucial.

The costs of surgery are not always incorporated when clinically considering distal or total gastrectomy; however, this is highly relevant for hospital management. In the LOGICA-trial, we had previously assessed the cost-effectiveness of D2-gastrectomy in detail: the mean total costs of D2-gastrectomy including costs associated with surgery (e.g., hospitalization, diagnostic modalities, complications, re-interventions, medication, emergency visits, rehabilitation and nursing homes, and productivity loss) noted €21,939 per distal and €31,583 per total D2-gastrectomy.^[42]^ This substantial cost-difference in favor of DG is mainly due to the lower complication rate, shorter hospitalization, and shorter operating time after DG versus TG, and may play a role in surgical decision making.

Since patients selected for DG differ in baseline from TG-patients by definition as described, this limits statistical comparison to some extent. However, the baseline differences are inherent to the indication per surgical procedure (DG/TG), and our findings consistently support DG in alignment with previously mentioned studies. Furthermore, several details of the Roux-en-Y reconstruction, including length of Roux-limbs and antecolic/retrocolic position and jejunal pouch formation and size, were not standardized in the LOGICA-trial, which could have resulted in (minor) differences in QoL-results between DG and TG.^[43,44]^ Strengths of this study are the LOGICA-randomization procedure that stratified for extent of resection (DG/TG) and hospital of surgical treatment, therefore minimizing selection bias, hospital reporting bias for postoperative complications, and differences in surgical outcomes due to hospital variation. In addition, the current secondary LOGICA-trial analysis is the first to assess surgical and oncological outcomes for Western gastric cancer patients in a prospective multicenter cohort incorporating neoadjuvant chemotherapy, and the first to report on quality of life after DG versus TG in a Western population. The reported outcomes may be considered high quality and representative for the Dutch population as 10 high-volume upper-GI centers participated.

In conclusion, Western gastric cancer patients selected for DG experienced fewer and less severe complications (overall, anastomotic leakage, pneumonia, atrial fibrillation), demonstrated quicker postoperative recovery, and reported substantial better quality of life, also after neoadjuvant chemotherapy, while oncological effectiveness after DG was safeguarded. Therefore, in selected patients where DG is oncologically feasible, DG should be preferred over TG. Alternatively, TG is safe and effective if adequate oncological control cannot be achieved with DG. To determine the optimal surgical strategy for each gastric cancer patient, it is crucial to individually balance radicality, surgical morbidity and quality of life.

### Supplementary Information

Below is the link to the electronic supplementary material.

Supplementary file1 (PDF 440 KB)

## Data Availability

Prof. dr. R. van Hillegersberf had full access to all trial data. The data are not publicly available for privacy reasons, and may be provided upon reasonable request.
