# Supplementary material for: Distal Versus Total D2-Gastrectomy for Gastric Cancer: a Secondary Analysis of Surgical and Oncological Outcomes Including Quality of Life in the Multicenter Randomized LOGICA-Trial
Source: J Gastrointest Surg. 2023 Jun 20;27(9):1812–24. doi: 10.1007/s11605-023-05683-z (PMC10511620; doi:10.1007/s11605-023-05683-z)
Supplement: Supplementary file 1 — Supplementary file1 (PDF 440 KB) [file 11605_2023_5683_MOESM1_ESM.pdf]

SUPPLEMENTARY MATERIAL

‘Distal versus total D2-gastrectomy for gastric cancer: a secondary analysis of surgical and oncological outcomes including quality of life in the multicenter randomized LOGICA-trial’

AUTHORS: Cas de Jongh[1] MD, Arjen van der Veen[1] MD, Lodewijk AA Brosens[1*] MD PhD, Grard AP Nieuwenhuijzen[2] MD PhD, Jan HMB Stoot[3] MD PhD, Jelle P Ruurda[1] MD PhD, Richard van Hillegersberg[1] MD PhD, on behalf of the LOGICA Study Group.

COLLABORATORS (LOGICA Study Group): Hylke JF Brenkman[1], Maarten F.J. Seesing[1], Misha DP Luyer[2], Jeroen EH Ponten[2], Juul JW Tegels[3], Karel WE Hulsewe[3], Bas PL Wijnhoven[4], Sjoerd M Lagarde[4], Wobbe O de Steur[5], Henk H Hartgrink[5], Ewout A Kouwenhoven[6], Marc J van Det[6], Eelco Wassenaar[7], P. van Duijvendijk[7], Werner A Draaisma[8], Ivo AMJ Broeders[8], Susanne S Gisbertz[9], Donald L van der Peet[10], Hanneke WM van Laarhoven[10*]

INSTITUTIONS AND AFFILIATIONS: [1]University Medical Center (UMC) Utrecht, Department of Surgery, Utrecht, The Netherlands. [1*]UMC Utrecht, Department of Pathology, Utrecht, The Netherlands. [2]Catharina Hospital Eindhoven, Department of Surgery, Eindhoven, The Netherlands. [3]Zuyderland Medical Center, Department of Surgery, Sittard, The Netherlands. [4]Erasmus UMC, Department of Surgery, Rotterdam, The Netherlands. [5]Leiden UMC, Department of Surgery, Leiden, The Netherlands. [6]ZGT Almelo, Department of Surgery, Almelo, The Netherlands. [7]Gelre Hospitals Apeldoorn, Department of Surgery, Apeldoorn, The Netherlands. [8]Meander Medical Center, Department of Surgery, Amersfoort, The Netherlands. [9]Amsterdam UMC, Location VUmc, Department of Surgery, Amsterdam, The Netherlands. [10]Amsterdam UMC, location AMC, Department of Surgery, Amsterdam, The Netherlands. [10*]Amsterdam UMC, Location AMC, Department of Medical Oncology, Amsterdam, Netherlands.

CORRESPONDING AUTHOR: Richard van Hillegersberg. Email: r.vanhillegersberg@umcutrecht.nl. UMC Utrecht, G04.228, Surgery Department; 3508 GA, Utrecht, The Netherlands. T: +31 (0)88-7558074. F: +31 (0)30-2541944. During review process: Cas de Jongh; c.dejongh@umcutrecht.nl.

SYNOPSIS: Distal D2-gastrectomy for gastric cancer resulted in less postoperative complications (overall, anastomotic leakage, pneumonia, atrial fibrillation), shorter hospitalization, quicker postoperative recovery and substantially better quality of life compared to total D2-gastrectomy, whereas radicality, nodal yield and overall survival were similar.

KEYWORDS: gastric cancer, gastrectomy, postoperative complications, quality of life, patient selection.

Supplementary Material – Index

Supplementary Table 1 – Factors associated with overall postoperative complications after distal versus total gastrectomy. *Page 3*

Supplementary Table 2 – Factors associated with radicality after distal versus total gastrectomy. *Page 4*

Supplementary Table 3 – Radicality after distal and total gastrectomy patients in various subgroups based on cT-stage, histological subtype and treatment with or without neoadjuvant chemotherapy. *Page 5*

Supplementary Table 4 – Quality of Life until 1 year after distal D2-gastrectomy using EORTC QLQ-C30 and STO-22 questionnaires. *Page 6*

Supplementary Table 5 – Quality of Life until 1 year after total D2-gastrectomy using EORTC QLQ-C30 and STO-22 questionnaires. *Page 7*

Supplementary Table 6 – Weight differences at 1 year follow-up postoperatively compared to the patients' preoperative baseline weight, stratified for distal and total gastrectomy. *Page 8*

Supplementary Figure 1 – Kaplan Meier curves for overall survival of distal versus total D2-gastrectomy. *Page 9*

Supplementary Table 1. Factors associated with overall postoperative complications after distal versus total gastrectomy.

**Entire cohort: n = 211 (100%)**	**Univariate**			**Multivariate**		
	**RR**	**95% CI**	***p-value***	**RR**	**95% CI**	***p-value***
**Age, per year**	1.03	1.01 – 1.05	**0.001**	1.02	1.00 – 1.04	**0.020**
**Any comorbidity (yes)**	1.05	0.70 – 1.58	0.810	1.04	0.67 – 1.59	0.859
**Tumor location**						
Proximal stomach	Ref	–	–	Ref	–	–
Middle stomach	0.50	0.48 – 1.17	0.199	0.83	0.52 – 1.34	0.455
Distal stomach	0.55	0.36 – 0.83	**0.005**	0.92	0.56 – 1.51	0.736
**Clinical T-stage**						
cT1	Ref	–	–	Ref	–	–
cT2	1.23	0.83 – 3.82	0.621	1.39	0.61 – 3.17	0.427
cT3	1.78	0.58 – 3.62	0.141	1.85	0.83 – 4.09	0.130
cT4	1.44	0.24 – 1.05	0.433	1.37	0.53 – 3.53	0.520
**Lauren classification**						
Intestinal type	Ref	–	–	Ref	–	–
Diffuse type	0.72	0.51 – 1.00	0.050	0.68	0.47 – 0.98	**0.041**
**Neoadjuvant chemotherapy (yes)**	0.67	0.48 – 0.92	**0.014**	0.71	0.48 – 1.04	0.076
**Type of resection**						
Total gastrectomy	Ref	–	–	Ref	–	–
Distal gastrectomy	0.46	0.34 – 0.64	**<0.001**	0.42	0.29 – 0.62	**<0.001**
**Surgical approach**						
Minimally invasive	Ref	–	–	Ref	–	–
Open	0.89	0.66 – 1.22	0.468	1.02	0.74 – 1.41	0.909

RR = relative risk. 95% CI = 95% confidence interval.
The multivariable regression analyses were performed using Poisson regression with robust error variance.

Supplementary Table 2. Factors associated with radicality after distal versus total gastrectomy.

Entire cohort: n = 211 (100%)	Univariate			Multivariate		
	**OR**	**95% CI**	***p-value***	**OR**	**95% CI**	***p-value***
**Type of resection**						
Distal gastrectomy	Ref	–	–	Ref	–	–
Total gastrectomy	5.93	1.23 – 28.62	**0.027**	2.98	0.49 – 18.23	0.238
**Tumor location**						
Proximal stomach	Ref	–	–	Ref	–	–
Middle stomach	1.31	0.14 – 12.40	0.812	1.66	0.15 – 18.49	0.678
Distal stomach	0.91	0.10 – 8.14	0.929	1.91	0.17 – 21.17	0.598
**Clinical T-stage**			0.158			
cT1–2	Ref	–	–	Ref	–	–
cT3–4	5.36	0.67 – 43.13	0.115	2.76	0.30 – 25.59	0.371
**Neoadjuvant chemotherapy (yes)**	1.43	0.30 – 6.96	0.656	1.03	0.19 – 5.74	0.973
**Maximum tumor diameter** (millimeter)	1.01	0.99 – 1.01	0.078	1.01	0.99 – 1.02	0.236
**Lauren classification**						
Intestinal type	Ref	–	–	Ref	–	–
Diffuse type	16.13	2.00 – 129.85	**0.009**	10.04	1.17 – 86.17	**0.035**

OR = odds ratio. 95% CI = 95% confidence interval.
These multivariable analyses were performed using multivariable binary logistic regression.

Supplementary Table 3. Radicality after distal and total gastrectomy patients in various subgroups based on cT-stage, histological subtype and treatment with or without neoadjuvant chemotherapy.

**Radicality in subgroups of distal gastrectomy patients N = 122 (100%)**					**Radicality in subgroups of total gastrectomy patients N = 89 (100%)**				
**Radicality; overall**					**Radicality; overall**				
**All patients**	**(n=122)**	**R0**	120	(98)	**All patients**	**(n=89)**	**R0**	81	(91)
		**R1**	2	(2)			**R1**	8	(9)
**Radicality; clinical T-stage**					**Radicality; clinical T-stage**				
**cT1–2**	**(n=57)**	**R0**	56	(98)	**cT1–2**	**(n=19)**	**R0**	19	(100)
		**R1**	1	(2)			**R1**	0	(0)
**cT3–4**	**(n=65)**	**R0**	64	(98)	**cT3–4**	**(n=70)**	**R0**	62	(89)
		**R1**	1	(2)			**R1**	8	(11)
**Radicality; histological subtype**					**Radicality; histological subtype**				
**Intestinal type**	**(n=85)**	**R0**	85	(100)	**Intestinal type**	**(n=45)**	**R0**	44	(98)
		**R1**	0	(0)			**R1**	1	(2)
**Diffuse type**	**(n=37)**	**R0**	35	(95)	**Diffuse type**	**(n=44)**	**R0**	37	(84)
		**R1**	2	(5)			**R1**	7	(16)
**Radicality; neoadjuvant chemotherapy**					**Radicality; neoadjuvant chemotherapy**				
**Yes**	**(n=86)**	**R0**	84	(98)	**Yes**	**(n=70)**	**R0**	64	(91)
		**R1**	2	(2)			**R1**	6	(9)
**No**	**(n=36)**	**R0**	36	(100)	**No**	**(n=19)**	**R0**	17	(90)
		**R1**	0	(0)			**R1**	2	(11)

Supplementary Table 4. Quality of Life until 1 year after distal D2-gastrectomy using EORTC QLQ-C30 (top) and STO-22 (bottom) questionnaires.

	Distal gastrectomy group at baseline (preoperative)		Distal gastrectomy at 6 weeks		Distal gastrectomy at 3 months		Distal gastrectomy at 6 months		Distal gastrectomy at 9 months		Distal gastrectomy at 1 year	
	Mean (±SD)		Mean (±SD)		Mean (±SD)		Mean (±SD)		Mean (±SD)		Mean (±SD)	
**Quality of life questionnaire (QLQ)-C30**												
Global health-related quality of life[1]	69	[22]	69	[23]	73	[20]	77	[18]	79	[19]	79	[18]
Functional scales[1]												
Physical functioning	81	[18]	71	[21]	77	[18]	80	[19]	80	[20]	83	[18]
Role functioning	72	[28]	60	[31]	71	[29]	80	[25]	81	[27]	81	[27]
Emotional functioning	80	[22]	78	[24]	83	[22]	85	[20]	85	[22]	88	[18]
Cognitive functioning	88	[17]	78	[24]	85	[20]	84	[22]	88	[17]	87	[17]
Social functioning	79	[26]	74	[27]	80	[24]	87	[20]	89	[20]	90	[19]
Symptom scales[2]												
Fatigue	32	[25]	44	[26]	36	[27]	26	[22]	27	[25]	24	[23]
Nausea and vomiting	10	[19]	16	[23]	12	[18]	6	[13]	7	[14]	6	[15]
Pain	14	[22]	27	[27]	17	[23]	17	[23]	17	[24]	12	[18]
Dyspnoea	17	[27]	20	[27]	15	[22]	17	[22]	17	[27]	10	[20]
Insomnia	26	[32]	33	[35]	26	[33]	24	[31]	22	[30]	22	[31]
Appetite loss	20	[31]	40	[37]	25	[31]	11	[23]	14	[24]	13	[23]
Constipation	12	[25]	16	[26]	10	[20]	7	[19]	8	[20]	6	[16]
Diarrhea	10	[22]	19	[29]	22	[29]	12	[22]	11	[21]	11	[22]
Financial difficulties	8	[23]	9	[20]	11	[23]	10	[21]	8	[17]	7	[16]
	Distal gastrectomy group at baseline (preoperative)		Distal gastrectomy at 6 weeks		Distal gastrectomy at 3 months		Distal gastrectomy at 6 months		Distal gastrectomy at 9 months		Distal gastrectomy at 1 year	
	Mean (±SD)		Mean (±SD)		Mean (±SD)		Mean (±SD)		Mean (±SD)		Mean (±SD)	
**Quality of life questionnaire STO-022**												
Functional scales[1]												
Body image	80	[30]	84	[26]	83	[28]	84	[29]	86	[25]	90	[21]
Symptom scales[2]												
Dysphagia	14	[24]	15	[23]	10	[16]	7	[14]	7	[15]	5	[12]
Pain	17	[23]	21	[22]	15	[19]	13	[17]	13	[18]	11	[16]
Reflux	15	[22]	17	[17]	13	[16]	11	[17]	12	[17]	8	[14]
Eating restrictions	21	[26]	33	[23]	22	[21]	16	[21]	15	[18]	14	[19]
Anxiety	36	[24]	35	[26]	30	[25]	25	[26]	22	[25]	19	[22]
Dry mouth	22	[29]	27	[30]	31	[32]	20	[29]	21	[29]	18	[24]
Taste	27	[34]	39	[37]	31	[33]	18	[28]	17	[25]	16	[25]
Hair loss	32	[41]	29	[38]	28	[37]	22	[35]	15	[32]	8	[23]

SD = standard deviation. [1] Scores range, 0-100: higher scores represent a better quality of life or functioning.
[2] Scores range, 0-100: higher scores represent more severe symptoms.

Supplementary Table 5. Quality of Life until 1 year after total D2-gastrectomy using EORTC QLQ-C30 (top) and STO-22 (bottom) questionnaires.

	Total gastrectomy group at baseline (preoperative)		Total gastrectomy at 6 weeks		Total gastrectomy at 3 months		Total gastrectomy at 6 months		Total gastrectomy at 9 months		Total gastrectomy at 1 year	
	Mean (±SD)		Mean (±SD)		Mean (±SD)		Mean (±SD)		Mean (±SD)		Mean (±SD)	
**Quality of life questionnaire (QLQ)-C30**												
Global health-related quality of life[1]	71	[20]	61	[21]	64	[20]	71	[18]	69	[19]	74	[18]
Functional scales[1]												
Physical functioning	82	[16]	68	[20]	73	[18]	79	[18]	77	[20]	77	[20]
Role functioning	75	[24]	54	[30]	61	[26]	73	[26]	74	[24]	72	[28]
Emotional functioning	80	[22]	79	[20]	80	[21]	83	[19]	82	[19]	83	[20]
Cognitive functioning	90	[19]	78	[25]	79	[23]	81	[21]	82	[19]	81	[19]
Social functioning	83	[24]	68	[26]	72	[25]	83	[20]	81	[20]	84	[19]
Symptom scales[2]												
Fatigue	27	[24]	48	[25]	41	[26]	33	[22]	34	[24]	32	[24]
Nausea and vomiting	6	[13]	24	[28]	23	[23]	17	[21]	14	[17]	9	[15]
Pain	11	[18]	25	[22]	19	[21]	18	[22]	22	[27]	18	[25]
Dyspnoea	17	[23]	19	[28]	19	[24]	14	[25]	15	[23]	14	[24]
Insomnia	16	[25]	28	[27]	28	[31]	28	[28]	26	[27]	20	[23]
Appetite loss	20	[31]	46	[36]	38	[34]	29	[30]	24	[28]	24	[30]
Constipation	10	[23]	12	[24]	6	[14]	8	[18]	9	[22]	9	[19]
Diarrhea	6	[14]	26	[30]	29	[31]	17	[24]	16	[20]	19	[24]
Financial difficulties	9	[21]	11	[23]	9	[19]	7	[17]	12	[20]	6	[15]

	Total gastrectomy group at baseline (preoperative)		Total gastrectomy at 6 weeks		Total gastrectomy at 3 months		Total gastrectomy at 6 months		Total gastrectomy at 9 months		Total gastre[illegible] at 1 year	
	Mean (±SD)		Mean (±SD)		Mean (±SD)		Mean (±SD)		Mean (±SD)		Mean (±SD)	
**Quality of life questionnaire STO-022**												
Functional scales[1]												
Body image	85	[30]	68	[33]	75	[29]	75	[28]	77	[30]	78	[31]
Symptom scales[2]												
Dysphagia	10	[20]	28	[24]	26	[25]	21	[25]	17	[20]	16	[16]
Pain	13	[18]	26	[19]	23	[19]	20	[16]	24	[22]	20	[20]
Reflux	10	[18]	21	[25]	24	[28]	19	[19]	24	[26]	19	[22]
Eating restrictions	16	[23]	42	[21]	38	[26]	33	[26]	31	[23]	28	[23]
Anxiety	35	[20]	40	[23]	39	[24]	35	[24]	37	[25]	32	[23]
Dry mouth	27	[33]	33	[35]	37	[31]	27	[27]	28	[28]	23	[27]
Taste	26	[32]	38	[35]	35	[30]	27	[29]	21	[29]	20	[24]
Hair loss	41	[39]	43	[41]	33	[38]	26	[34]	20	[33]	13	[26]

SD = standard deviation. [1] Scores range, 0-100: higher scores represent a better quality of life or functioning.
[2] Scores range, 0-100: higher scores represent more severe symptoms.

Supplementary Table 6. Weight difference at 1 year follow-up postoperatively compared to the patients' preoperative baseline weight, stratified for distal and total gastrectomy.

		Entire cohort<br>n = 115 (100%)		Distal gastrectomy<br>n = 73 (100%)		Total gastrectomy<br>n = 42 (100%)		*p*-value
**Weight difference, in kg**	*median [IQR]*	-6 kg	[-1 to -11]	-4 kg	[+1 to -8]	-10 kg	[-5 to -15]	***<0.001***
**Weight difference**								***0.003***
Weight loss	> 2 kg	73	(64)	38	(52)	35	(83)	
Similar weight	± 2 kg	31	(27)	25	(34)	6	(14)	
Weight increase	> 2 kg	11	(10)	10	(14)	1	(2)	

A negative weight difference indicates weight loss compared to the preoperative baseline weight.
A positive weight difference indicates a weight increase in comparison to the preoperative baseline weight.

Supplementary Figure 1. Kaplan Meier curves for overall survival of distal versus total D2-gastrectomy.

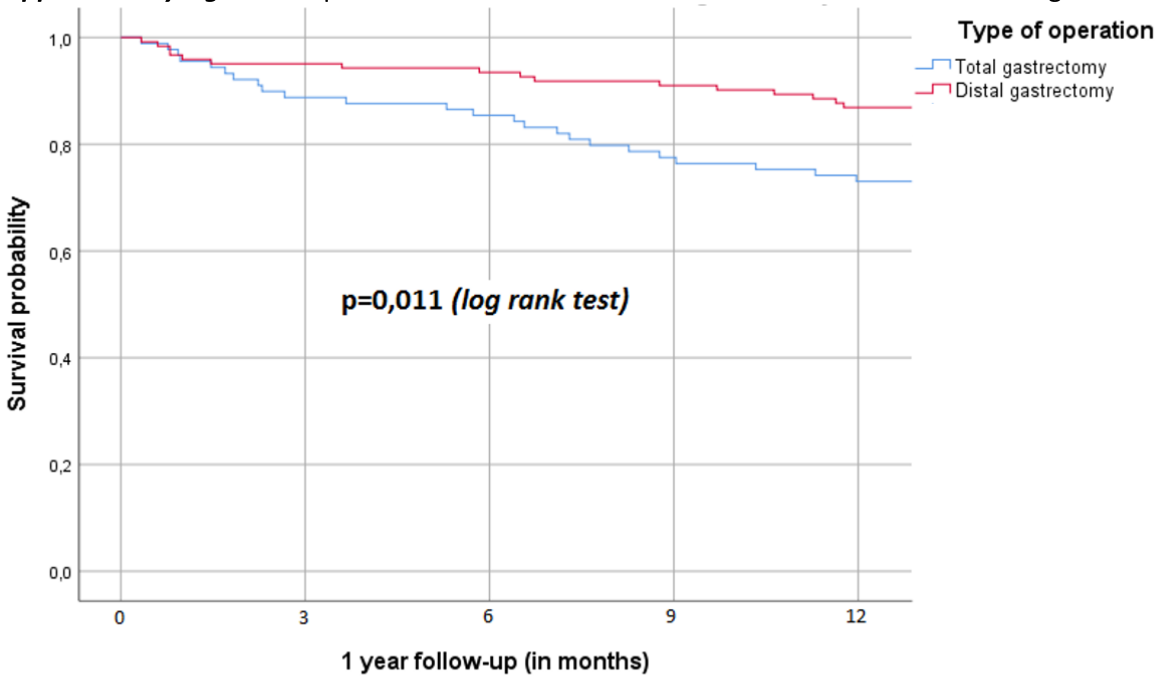

Numbers at risk

	0	3	6	9	12
**Total gastrectomy**	89	79	76	69	65
**Distal gastrectomy**	122	116	114	111	106

Time in months

Please note that these Kaplan Meier curves are univariate and cannot incorporate the significant baseline differences in age, comorbidities, tumor location, clinical T-stage and histological subtype between the distal and total gastrectomy patient groups. After adjusting for these baseline differences in multivariable Cox Proportional Hazards regression analyses (Table 5), overall survival for patients undergoing distal versus total gastrectomy was <u>*not*</u> *significantly different (p=0.084).*
